# Supplementary material for: An Optical Sensor with Polyaniline-Gold Hybrid Nanostructures for Monitoring pH in Saliva
Source: Nanomaterials (Basel). 2017 Mar 17;7(3):67. doi: 10.3390/nano7030067 (PMC5388169; doi:10.3390/nano7030067)
Supplement: Supplementary file 1 [file nanomaterials-07-00067-s001.pdf]

Nanomaterials **2017**, *7*, 67; doi:10.3390/nano7030067

Supplementary Materials: An Optical Sensor with Polyaniline-Gold Hybrid Nanostructures for Monitoring pH in Saliva

Chongdai Luo, Yangyang Wang, Xuemeng Li, Xueqin Jiang, Panpan Gao, Kang Sun, Jianhua Zhou, Zhiguang Zhang and Qing Jiang

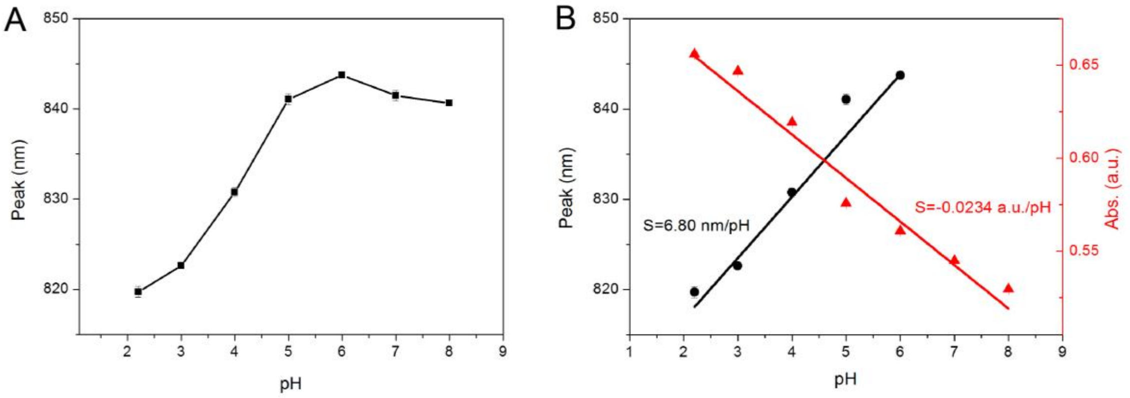

Figure S1. (**A**) The peak shift of PANI-GNPs-glass in different pH media; (**B**) two pH linear ranges of this PANI-GNPs-glass biosensor based on: the peak shift of PANI-GNPs-glass (black) and absorbance at 785 nm (red).

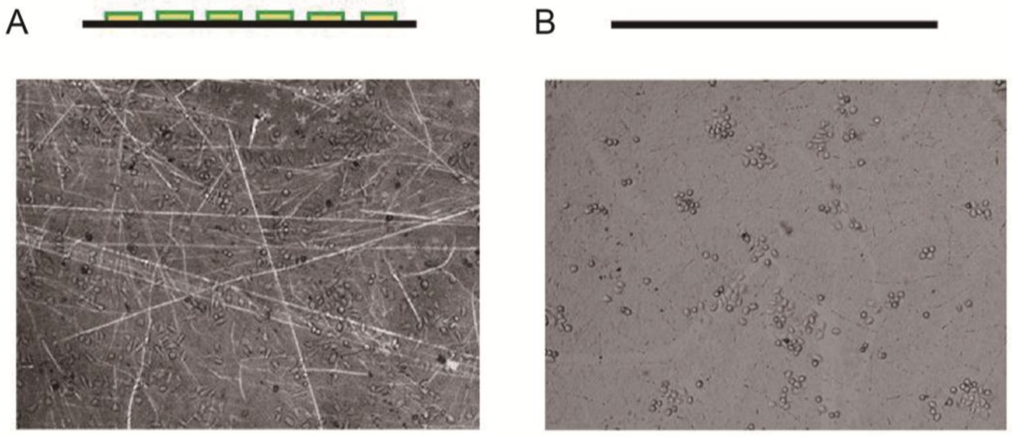

Figure S2. The viability of A549 cell on (**A**) GNPs-PANI-glass sensing substrate and (B) On glass.
